# Nucleic acid-based therapeutics for the treatment of central nervous system disorders

**DOI:** 10.3389/fgene.2023.1250276

**Published:** 2023-08-16

**Authors:** Robyn McCartan, Olga Khorkova, Claude-Henry Volmar, Claes Wahlestedt

**Affiliations:** ^1^ Center for Therapeutic Innovation, University of Miami Miller School of Medicine, Miami, Florida, United States; ^2^ Department of Psychiatry and Behavioral Sciences, University of Miami Miller School of Medicine, Miami, Florida, United States; ^3^ OPKO Health, Miami, Florida, United States

**Keywords:** siRNA, ASO, oligonucleotide, blood brain barrier, drug discovery

## Abstract

Nucleic acid-based therapeutics (NBTs) are an emerging class of drugs with potential for the treatment of a wide range of central nervous system conditions. To date, pertaining to CNS indications, there are two commercially available NBTs and a large number of ongoing clinical trials. However, these NBTs are applied directly to the brain due to very low blood brain barrier permeability. In this review, we outline recent advances in chemical modifications of NBTs and NBT delivery techniques intended to promote brain exposure, efficacy, and possible future systemic application.

## 1 Introduction

CNS disorders comprise conditions with diverse etiologies ranging from neurodegenerative diseases and mental health disorders to epilepsy and stroke. Despite recent advancements in pharmacology and neuroscience, the therapeutic options for many CNS disorders remain limited. Factors contributing to the limited availability of treatment options include the complexity of the brain, the diverse genetic factors contributing to disease, and the presence of the blood-brain barrier. Of these factors, the presence of the blood brain barrier remains the main challenge of NBT delivery to the brain.

An emerging class of therapeutics that holds significant potential for the treatment of CNS conditions is nucleic acid-based therapeutics (NBTs). Thirty years have passed since the first successful *in vivo* application of NBTs, specifically antisense oligonucleotides (ASOs), in the brains of rats and mice (Wahlestedt et al., 1993a; Wahlestedt et al., 1993b; Standifer et al., 1994). Today, the constantly expanding range of NBT modalities includes not only ASOs but also short interfering RNAs (siRNAs), full-length therapeutic mRNAs, gene therapy constructs, nucleic acid-programmable genome and RNA-editing enzymes (CRISPR-Cas13, ADARs) as well as various vectorized constructs delivering single or multiple NBTs (Zogg et al., 2022). NBTs allow for highly specific and efficient targeting of disease-relevant genes and have benefits over traditional small molecule drugs, such as limited off-target effects and high speed of pharmaceutical development due to rapid design and synthesis of NBTs (Kim, 2022). These qualities along with the ability of NBTs to target specific alleles, isoforms, and point mutations creates new possibilities for personalized medicine in the field of rare diseases (Khorkova et al., 2021). Common chemical structures of different NBT types favors class-wide application of newly discovered chemistries and delivery techniques. Furthermore, NBTs represent an efficient modality for accessing a recently discovered set of novel therapeutic targets related to regulatory long non-coding RNA (lncRNA), which vastly increases the range of treatable diseases. In particular, natural antisense transcripts (NATs), a subclass of lncRNA, prove as promising therapeutic targets due to their role in gene expression and RNA-based regulatory networks (Khorkova et al., 2023).

In rodents and non-human primates *in vivo* administration of NBTs has proven successful in ameliorating pathological features of various CNS disorders including focal ischemia, leptomeningeal amyloidosis, MECP2 duplication syndrome, Dravet syndrome, and Angelman syndrome (Khorkova and Wahlestedt, 2017). Given the success of these *in vivo* studies, NBTs have great promise for efficacy in a clinical setting. This review will focus on the potential of ASOs and siRNAs, two NBT modalities with several drugs already in the clinic, as treatment options for CNS disorders. Topics highlighted in this review include advancements in overcoming obstacles such as nuclease degradation and entrapment in endosomal compartments, developments in modifications and nanotechnology allowing for BBB penetration, progress in understanding the pharmacokinetics and pharmacodynamics of NBTs, and developments in routes of administration.

## 2 NBTs: ASOs and siRNA

### 2.1 ASOs

ASOs typically consist of a single strand of approximately 20 nucleotides that are complementary to a target RNA transcript or genomic sequence. ASOs interact with their target strands via Watson-Crick base pairing, leading to gene expression changes (Dhuri et al., 2020). One of the mechanisms by which ASOs can change gene expression is through the recruitment of the enzyme RNAse H, which leads to the degradation of mRNA transcripts and thus a decrease in gene expression (Lai et al., 2020). Other mechanisms of action of ASOs include inducing steric hindrance that interferes with the function of transcriptional or translational machinery. Modulation of splicing machinery by ASOs can be used to correct the reading frame of mutated transcripts leading to either exon skipping or exon inclusion (Amanat et al., 2022).

### 2.2 siRNAs

siRNAs are synthetic double-stranded RNA molecules of approximately 20–25 nucleotides that function by exploiting the RNA interference (RNAi) pathway, a cellular pathway that regulates gene expression through the use of endogenous double-stranded RNA molecules (microRNAs or miRNAs) to degrade specific mRNA or lncRNA transcripts (Traber and Yu, 2023). The mechanism of action of siRNA is highly specific and efficient, making it a powerful tool for modulating gene expression.

### 2.3 NBT chemistry: Internal chemical modifications

Unmodified DNA and especially RNA molecules are highly vulnerable to degradation by endogenous nucleases (Gagliardi and Ashizawa, 2021). To maintain structural integrity and functionality *in vivo*, therapeutic ASOs and siRNAs must undergo chemical modifications that promote stability and cellular uptake. Internal chemical modifications of NBTs can be divided into three categories: sugar modifications, backbone modifications, and base modifications.

### 2.4 Sugar modifications

**FIGURE 1:**
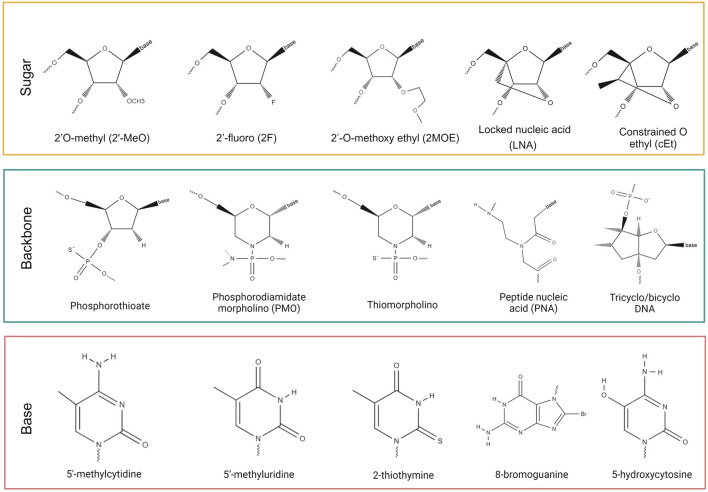
Chemical modifications of NBTs.

Common sugar modifications include 2′O-methyl (2′-MeO), 2′-fluoro (2F), 2′-O-methoxy ethyl (2MOE), locked nucleic acids (LNA) and constrained 2′-O ethyl (cEt) (Figure 1) (Prakash, 2011). Sugar modifications are known to increase NBT binding affinity to target RNA/DNA, stability against nucleases, and limit immune response activation (Shen et al., 2019).

### 2.5 Backbone modifications

Backbone modifications range from single atom substitutions, as in phosphorothioate (PS) backbones, to complete replacement of natural phosphodiester backbone with phosphorodiamidate morpholino (PMO), thiomorpholino, tricyclo/bicyclo DNA, peptide nucleic acid (PNA) and other groups (Figure 1) (Crooke et al., 2020b). Most modified backbones increase hydrophobicity of NBTs, which improves their pharmacokinetic (PK) properties by facilitating interactions with proteins and lipids. Incorporation of neutral backbone linkages, such as methoxylpropyl, isopropyl and isobutyl phosphonates and O-isopropyl and O-tetrahydrofuranosyl phosphotriesters, formacetal and C3-amide, was shown to improve therapeutic index, with final ASO activity depending on the size, hydrophobicity, and RNA-binding affinity of the linkage (Vasquez et al., 2022). In rare cases ASOs activate TLR9 signaling in a sequence dependent-manner. This effect can be alleviated by using two mesyl phosphoramidate linkages within the PS ASO gap (Pollak et al., 2023).

### 2.6 Base modifications

Base modifications, such as 5′-methylcytidine, 5′-methyluridine, 2-thiothymine, 8-bromoguanine and 5-hydroxycytosine are known to reduce off-target cleavage by ASOs and inhibit immune system activation (Figure 1) (Song et al., 2022).

Although many of these modifications do not support the mechanisms of action of ASOs and siRNA through interfering with RNAseH and RNAi activity, respectively, Although many of these modifications do not support the mechanisms of action of ASOs and siRNA through interfering with RNAseH or RNAi activity, if modified oligonucleotides are positioned to form so called gapmers, with a modification-free 8–12 nucleotide gap in the center flanked by modified nucleotide “wings”, they can be used to engage these mechanisms (Brooke et al., 2021). Non-gapmer modified oligonucleotides (mixmers) can also be used in steric hindrance applications (Yoshida et al., 2023).

## 3 External modifications of NBTs

### 3.1 Di-valent siRNA scaffolds

The number of PS backbone modifications helps promote systemic and CNS distribution of NBTs, with more PS modifications resulting in better distribution (Crooke et al., 2020a). Specifically, ASOs containing a PS content of 80% have proven especially successful in regard to distribution and efficacy (Sewing et al., 2017; Valenzuela et al., 2023). However, the presence of PS modifications above 40% in siRNA can negatively impact the efficacy of RNA induced silencing complex (RISC) loading and promote off target effects and toxicity (Biscans et al., 2020; Halloy et al., 2022).

A developing method to increase PS content while retaining efficacy and reducing toxicity of siRNA are di-valent siRNA scaffolds (di-siRNA). A di-siRNA consists of two siRNAs connected by a linker. The administration of di-valent siRNAs *in vivo* has resulted in increased brain distribution, target silencing, and duration of silencing (Alterman et al., 2019; Conroy et al., 2022; O'Reilly et al., 2023). Specifically, a single high dose of 475ug of di-siRNA targeting the HTT gene was administered to BACHD-ΔN17 mice via intracerebroventricular (ICV) injection and resulted in significant HTT silencing and guide strand accumulation in key brain regions such as the cortex and striatum for up to 6 months (Alterman et al., 2019). Similarly in non-human primates (NHP), a single 25 mg dose of the same di-siRNA administered via ICV injection resulted in significant HTT silencing and guide strand accumulation throughout the brain at 1 month. Importantly, there were limited off target effects and no discernible toxicity (Alterman et al., 2019). An additional study found that when Msh3 was targeted with a di-siRNA, CAG-repeat expansion was blocked in the striatum of two different Huntington’s disease mouse models for up to 4 months and no off target effects were observed (O'Reilly et al., 2023).

Given the demonstrated success of di-siRNAs in distributing throughout the brain, knocking down targets for sustained periods, and the lack of off target effects and toxicity, di-siRNAs hold great promise in the field of NBTs. Furthermore, the proven capability of linking two NBTs while preserving their effectiveness has paved the way for linking NBTs with distinct targets. This approach can be beneficial for conditions with multiple therapeutic targets, as it enables the development of an NBT that can simultaneously address two distinct targets.

### 3.2 Bioconjugation

Bioconjugation of siRNAs or ASOs to different moieties such as peptides, antibodies, lipids, or sugars can be done to promote targeted delivery, bioavailability, and cellular uptake (Craig et al., 2018). Bioconjugation can also help address *in vivo* delivery challenges such as promoting endosomal escape with cell-penetrating peptides (CPPs). For example, a study examining the effectiveness of the CPP HA2-ApoE (130-150) showed promising results in increasing the levels of functional SMN2 mRNA in patient-derived fibroblasts and in the SMN2 transgenic mouse model of spinal muscular atrophy (SMA) when administered via tail vein injection at a dose of 8 mg/kg (Dastpeyman et al., 2021). The HA2-ApoE residues (130-150) are known to interact with low density lipoprotein receptors (LDLRs), which are present on brain endothelial cells and support the transport of lipoproteins across the BBB.

#### 3.2.1 Cholesterol conjugation

Cholesterol conjugation is an approach that can increase lipophilicity to enhance BBB penetration. Heteroduplex oligonucleotides conjugated to cholesterol can reach the CNS via intravenous (IV) injection in mice and rats and suppress Malat1 expression by up to 90% (Nagata et al., 2021). Additionally, the 2′-O-hexadecyl (C16) lipophilic modification of siRNA has shown great efficacy preclinically in mouse and NHP models. In CVN-AD mice, a 120 µg dose of C16 conjugated siRNA targeting APP administered via ICV injection led to a 75% reduction of APP mRNA at 30 days, reduced associated pathological makers, and improved behavioral deficits. Intrathecal (IT) injection of 60 mg of C16 conjugated siRNA also targeting APP in NHPs resulted in an 80% knockdown of APP in the brain, with a 75% knockdown sustained for 2.5 months (Brown et al., 2022). This preclinical success has led Alnylam and Regeneron to run a phase I study testing the C16 conjugated siRNA in 20 early onset Alzheimer’s disease patients. Preliminary results from this study has shown that IT injection of the C16 conjugate leads to a reduction of APP and its metabolite by 70% in the CSF of patients. Cognitive function was not evaluated (Alnylam Pharmaceuticals, 2023).

#### 3.2.2 Conjugations targeting transferrin receptor family

Conjugation of oligonucleotides to antibodies has also shown success regarding brain administration. When an ASO targeting the SMN2 gene was conjugated to an antibody targeting the murine transferrin receptor and administered systemically at a dose of 50 mg/kg in a hSMN2-transgenic mouse model, therapeutic levels of SMN2 splicing demonstrated by a 2-fold increase of FLSMN2 expression were reached in the mouse brain (Hammond et al., 2022).

Given the widespread presence of TfR1 on brain endothelial cells, facilitating drug uptake into the brain via TfR1-mediated transcytosis has been a mechanism of interest. A recent study in preprint has demonstrated successful ASO brain administration through the targeting of TfR1 in mice and NHPs (Barker et al., 2023). In this study, ASOs were conjugated to a transport vehicle (TV) that was engineered to have a binding affinity for TfR1. The TV used was a huIgG containing an engineered Fc domain. When administered systemically at a dose of 2.4 mg/kg, the ASO-TV complex targeting Malat1 proved successful in penetrating the BBB and distributing ASO throughout the brain of TfR^mu/hu^ KI mice (Barker et al., 2023). A significant and sustained level of Malat1 knockdown was demonstrated in the brain of both mice and NHPs.

siRNAs can also utilize the presence of the transferrin receptor family on the BBB to aid in brain administration. Specifically, when administered in BALB/c mice three times via IV injection at a dose of 30 mg/kg, siRNA targeting the NOX4 gene was able to bypass the BBB when conjugated to a peptide that interacts with the melanotransferrin receptor (Eyford et al., 2021). The peptide, MTfp, was demonstrated to act as a “nanomule” allowing the siRNA to reach the brain and therapeutically knock down levels of NOX4.

#### 3.2.3 Conjugations targeting GLUT1 transporter

Through targeting the GLUT1 transporter, carrier-mediated transcytosis can be exploited to facilitate drug transfer across the BBB. GLUT1 is present on the surface of the plasma membrane of brain capillary endothelial cells (BCEC). When blood glucose levels rise after fasting, GLUT1 undergoes transcytosis and travels from the apical to the basal side of BCEC membranes (Koepsell, 2020). Nanoparticles modified with a glucose moiety can interact with GLUT1 when it is located on the apical side, and once transcytosis occurs due to changes in blood glucose levels, the nanoparticle can travel along with GLUT1 to the basal side and be released into the brain. Taking advantage of this mechanism, a study showed that after just a single IV injection of 100 µg of an ASO contained within a glucose-coated polymeric nanocarrier, a 40% knockdown of the target Malat1 was obtained in both the cortex and hippocampus (Min et al., 2020). Furthermore, a study that developed a glycosylated polymeric siRNA nanomedicine known as Gal-NP@siRNA showed that systemic administration of 1 mg/kg of the glycosylated siRNA led to lower expression of the target gene and improvement of cognitive decline in the APP/PS1 mouse model of Alzheimer’s disease (Zhou et al., 2020).

### 3.3 Nanoparticles

Success in improving the bioavailability of NBTs in the brain after systemic administration via chemical modification is further advanced through the use of nanotechnology and nanoparticles. To effectively deliver NBTs to the brain as well as other tissues, nanoparticles must protect against degradation in the blood before reaching the BBB and must facilitate the crossing of BBB.

#### 3.3.1 Polymeric nanoparticles

Polymeric nanoparticles are an attractive option as their composition and surface properties can be modified to control the drug release rate and promote cell type-specific delivery (Amulya et al., 2023). Polymeric nanoparticles comprise a lipid with dissolved drug cargo surrounded by a biodegradable polymeric shell, which can be engineered to control cargo release. Alternatively, they can be formed by a continuous polymeric network containing the drug (Marangoni and Menezes, 2022). However, a disadvantage of polymer-based delivery systems is that they have poor NBT transfer efficiency and are rapidly cleared. A polyethylene glycol (PEG) coating can increase circulation time and reduce nonspecific cellular uptake (Shi et al., 2021). A recent study has demonstrated that modulating the surface coating and surface density of polymeric nanoparticles can augment the active transport of the nanoparticle across the BBB (Li et al., 2021). The study showed that 50 nmol/kg of a systemically administered poly-lactic-co-glycolic acid (PLGA)-based nanoparticle coated in polysorbate 80 (PS 80) increased brain penetration threefold compared to traditional PEG-coated nanoparticles in the mouse brain. Additionally, the PS 80 nanoparticle containing tau-targeting siRNA led to a 50% decrease in tau expression in the brain after systemic administration (Li et al., 2021). Use of 3D printing in polymeric nanoparticle production can improve their mechanical properties, shapes and release profiles as well as improve the quality and scalability of the nanoparticle manufacturing process (Kutlehria et al., 2022).

#### 3.3.2 Lipid-based nanoparticles

Lipid-based nanoparticles (LNPs) have a lipid bilayer shell and lipid core matrix that is stabilized by emulsifiers. The lipid bilayer encapsulates the NBT and protects it from degradation, ultimately allowing for entrance into cells. Although LNPs are too large to cross the BBB, they are capable of targeting the brain when administered via ICV or IT injection (Bost et al., 2021). One way that LNPs can be modified to cross the BBB is through the creation of neurotransmitter-derived lipidoids (NT-lipidoids). The hybrid structure of NT-lipidoids can interact with receptors on the BBB and facilitate transport. NT-lipidoids containing an ASO targeting tau successfully bypassed the BBB when administered intravenously at a dose of 1 mg/kg and led to the knockdown of tau mRNA and protein (Ma et al., 2020).

#### 3.3.3 Inorganic nanoparticles

Another category is inorganic nanoparticles, which includes gold nanoparticles (AuNPs). Although highly charged AuNPs can activate an immune response in the brain, surface modifications can be added to help alter the charge (Kus-Liskiewicz et al., 2021). AuNPs usually consist of an inner inorganic layer that is surrounded by a nonpolar organic layer that helps stabilize the core and attaches to the NBT (Kasina et al., 2022). Surface modifications can be added to aid with targeting, while the small size of AuNPs helps with crossing the BBB. Coating of an AuNP with bioengineered brain-targeting exosomes has aided in brain uptake. A study utilizing this surface modification showed accumulation of the AuNP in the brain after systemic administration of 200 µL in mice (Khongkow et al., 2019).

#### 3.3.4 Exosomes

Lastly, exosomes have also been explored as a potential delivery system to the CNS. They do not stimulate an immune response and have an advantage in that they can carry more than one drug simultaneously (Cecchin et al., 2023). Since exosomes are surrounded by a lipid bilayer, they are membrane-permeable and can cross the BBB. Drug-containing exosomes have been demonstrated to reach the CNS via IV administration when conjugated with the rabies virus glycoprotein (Sun et al., 2022). However, loading drugs into exosomes remains a challenge (Bost et al., 2021). One way this limitation has been overcome is through the reprogramming of hepatocytes to transcribe siRNA and package it into exosomes (Zhang et al., 2021). The siRNA-containing exosomes formed by the hepatocytes are tagged with the rabies virus glycoprotein and exploit the endogenous exosome-circulating system to bypass the BBB. This technology has proven effective in reducing levels of expression of the mutated Huntington’s disease (HD) gene in the cortex and striatum in three different mouse models of HD including N171-82Q, BACHD, and YAC128 (Zhang et al., 2021).

## 4 NBT uptake

### 4.1 BBB uptake mechanisms

There are several mechanisms by which NBTs can cross the BBB, including paracellular transport, carrier-mediated transport, receptor-mediated transport, and absorptive-mediated transport.

Paracellular transport occurs through the junctions between the brain endothelial cells or in areas with weak BBB integrity which allows for the passage of small hydrophilic compounds. In regard to NBT delivery, paracellular transport across the BBB is relatively inefficient. However, hyperosmotic solutions, such as those containing mannitol, can be administered systemically to alter the osmotic pressure of endothelial cells at the BBB, which allows the tight junctions to open, and facilitates paracellular transport of NBTs (Chu et al., 2022).

Carrier-mediated transport is another mechanism that allows NBTs to cross the BBB using solute carriers. Specifically, the molecules bind to carrier proteins present on the membrane, allowing the molecule to become internalized and move from one side of the BBB to the other due to a concentration gradient (Yazdani et al., 2019).

Receptor-mediated transport is another possible mechanism, in which an NBT is conjugated with a ligand specific to a receptor present in brain endothelial cells. One method by which receptor-mediated transport is exploited for drug delivery to the brain is targeting the transferrin receptor (Tfr1) abundant on brain endothelial cells (Thomsen et al., 2022).

Lastly, absorptive-mediated transport is a mechanism by which cationic molecules can bind to the luminal surface of endothelial cells and be taken up by the cell and transported across the BBB (Knox et al., 2022). An example of this is nanoparticles that interact with lipid rafts of the plasma membrane. Lipid rafts are negatively charged, which promotes interaction with positively charged nanoparticles or nanoparticles being carried by positively charged serum molecules (Hald Albertsen et al., 2022).

### 4.2 Cellular uptake mechanisms

It is proposed that cellular uptake of ASOs predominately occurs through receptor-mediated endocytosis via clathrin and caveolin-dependent mechanisms (Migliorati et al., 2022). Multiple pathways for ASO internalization exist, and they often involve surface receptors such as integrins, GPCRs, and scavenger receptors (D’Souza et al., 2023). Once internalized, the ASOs can be trafficked from early endosomes to late endosomes and then to lysosomes (Holm et al., 2022).

Common uptake mechanisms of siRNAs are similar to that of ASOs and include clathrin- and caveolin-mediated endocytosis (Juliano et al., 2012). Intracellular trafficking of siRNA follows a similar pathway to ASOs and involves traveling through the early and late endosomes. Both ASOs and siRNA must escape the late endosome to ensure entry to the cytoplasm to interact with their target pathways (Holm et al., 2022; Dowdy, 2023).

As a result, although oligonucleotides are readily taken up and retained by the cells, they are largely sequestered in these subcellular membranous structures, with only a small proportion available for productive regulation of gene expression. NBTs were shown to escape sequestration mainly from pre-lysosomal compartments, during multiple vesicle budding and fusion events (Gokirmak et al., 2021). The exact mechanisms of endosomal escape have not been elucidated yet and methods to improve this process are under development (Roberts et al., 2020; Gokirmak et al., 2021).

Multiple methods to increase the probability of endosomal escape, and thus the bioavailability of NBTs, have been proposed. For example, a non-toxic synthetic sphingosine analog SH-BC-893 inactivated the small GTPase ARF6, which inhibited lysosomal fusion and trapped endocytosed oligonucleotides in pre-lysosomal compartments. This resulted in an up to 200-fold increase in activity of ASOs and siRNAs (Finicle et al., 2023).

Furthermore, conjugation of a cell-penetrating peptide HA2-ApoE (131-150) containing several endosomal escape domains to nusinersen significantly increased the level of full-length SMN2 in the brain and spinal cord of SMN2 transgenic mice after systemic administration (Dastpeyman et al., 2021).

Identifying genes involved in productive ASO release may help in designing therapeutic interventions to increase it. Overexpression of GOLGA8, a novel protein localized predominantly to the trans-Golgi and plasma membrane increased uptake and activity of splice modulating and RNase H1-dependent ASOs (McMahon et al., 2023).

However, cellular uptake is only a small part of the challenges that complicate NBT delivery *in vivo*. After IV or subcutaneous injection, NBTs are subject to nuclease degradation in blood and interstitial tissues, sequestration by plasma proteins, phagocytosis by specialized blood, liver and kidney cells and renal clearance. Furthermore, to reach the target cells they must pass through the endothelial barrier and/or BBB to reach the CNS (Roberts et al., 2020; Gokirmak et al., 2021).

## 5 NBT distribution

**FIGURE 2:**
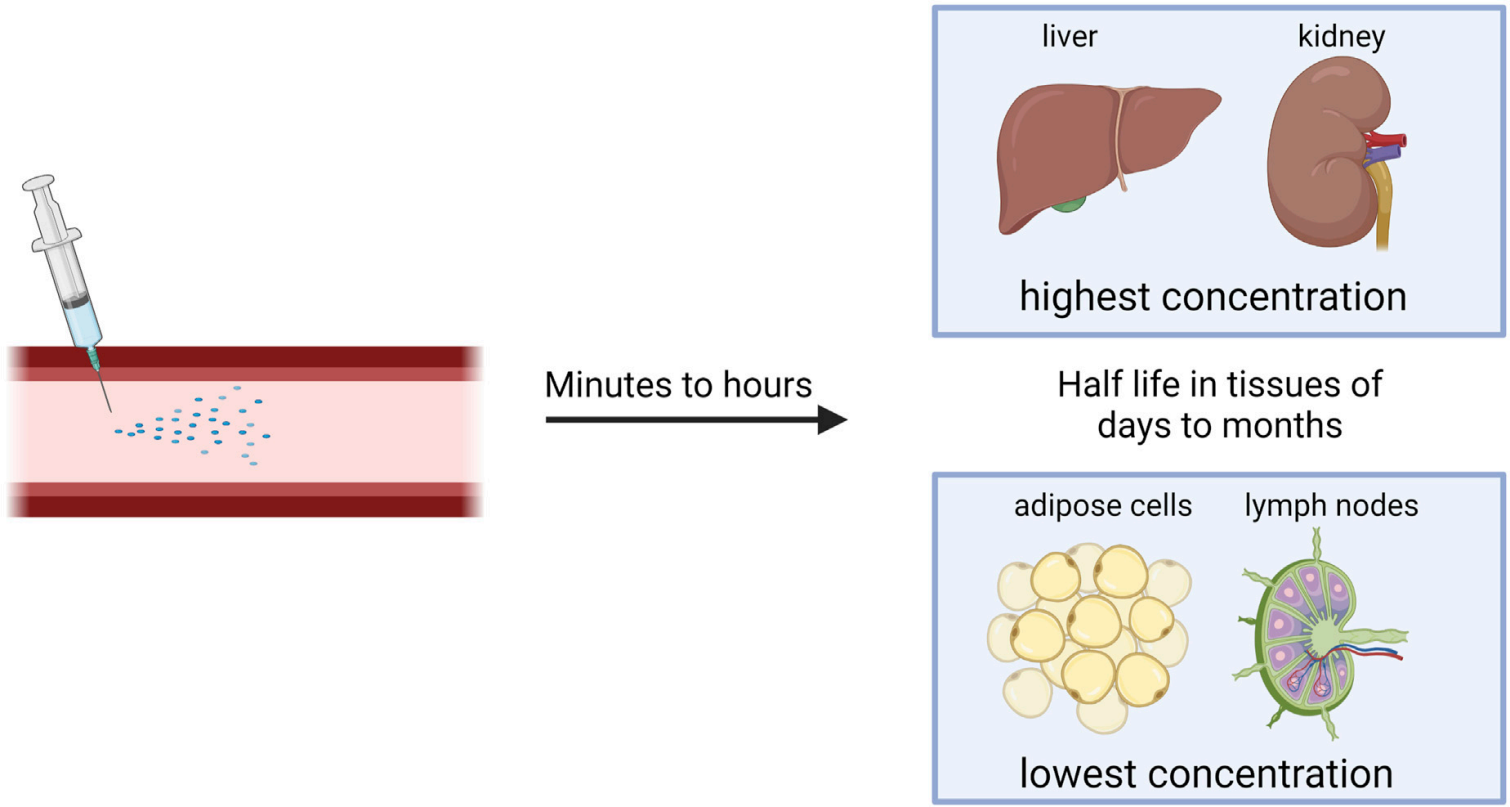
Distribution of NBTs after systemic injection.

After systemic or subcutaneous administration, tissues with highest ASO concentration are liver and kidney, but ASOs also accumulate in bone marrow, the cell body of adipocytes, and lymph nodes (Figure 2) (Takakusa et al., 2023). The pharmacokinetic properties of naked ASOs are enhanced by the presence of a phosphorothioate backbone, which allows for the binding of serum proteins that serve as molecular shuttles for the drug (Vasquez et al., 2022).

The transfer of ASOs or siRNA from blood to tissues occurs in minutes to hours after administration (Figure 2), and 80% of the plasma concentration can be excreted through kidneys within 24 h. After initial quick clearance, low levels of ASOs persist in plasma with a half-life of 2–8 weeks, possibly due to slow release from interstitial space and intracellular depots. siRNA is eliminated from the plasma within minutes to hours, but once within tissues, it has a half-life of days to months (Figure 2) (Jeon et al., 2022). To increase stability in the plasma, albumin-binding aptamer chimeras have shown success (Rosch et al., 2022). This is due to the long half-life and high concentrations of albumin within the plasma.

After direct CNS administration, ASOs quickly disperse over all brain regions with a gradient of concentration starting from the point of injection. Single nucleus transcriptomics have demonstrated that in mice and NHPs treated with ASOs against Prnp and Malat1 target knockdown was observed in every cell type, and in most individual cells, as opposed to large effect in a small number of cells. The knockdown duration was shorter in microglia than in neurons and lasted for up to 12 weeks after injection. In NHPs, the level of PRNP knockdown in CNS adequately reflected the level of effects in CNS cells (Mortberg et al., 2023).

### 5.1 NBT pharmacokinetics and pharmacodynamics

Novel PK and pharmacodynamic (PD) properties of NBTs complicate toxicology studies and clinical trial design. Predictive power of animal models for different NBT modalities is also poorly studied at this time. As the number of clinical studies of NBTs and the numbers of patients dosed in each study increase, PK models can be developed. Population PK models are useful in determining therapeutic windows and dosage recommendations for NBTs. These models integrate available PK data to identify factors that can affect individual drug exposure. Based on data collected from 750 participants from five clinical studies of an IT-administered ASO tominersen (Table 2), CSF PK could be modeled using a three-compartment design with first-order transfer from CSF to plasma, while a three-compartment model with first-order elimination from plasma was needed for plasma PK. Significant covariates for CSF clearance included baseline total CSF protein, age, and levels of antidrug antibodies (ADAs). Clearance from plasma covaried with body weight (Yamamoto et al., 2023).

A whole-body mechanistic model of ASO distribution in after IT injection based on NHP data has been proposed. Physiological parameters and kinetic uptake rates were set using PK data for 2′-MOE PS gapmers in cynomolgus monkeys. The model adequately described spinal transport of an IT-administered ASO due to pulsation-enhanced mixing, advection and diffusion, as well as ASO transfer to systemic circulation, CSF and peripheral tissue compartments. The model considered six anatomical regions: spinal CSF, spinal cord tissue, cranial CSF, cranial tissue, blood and peripheral tissues. Cranial tissue was subdivided into compartments corresponding to pons, hippocampus, cerebellum, and cortex. Spatiotemporal distribution of ASO in these compartments as a function of injection volume and duration revealed that higher infusion volume can enhance drug dispersion along the spinal axis, while shorter duration of injection results in better cranial delivery compared to a long infusion of the same volume. The model uses physics-based scaling laws to assess subject-specific variations (e.g., adult-child) or enable translation of data across species (Linninger et al., 2023).

Furthermore, in order to better understand NBT distribution, there is a need for microscopic approaches. Techniques such as fluorescence imaging, mass imaging, and immunohistochemistry are emerging as promising methods for tracking these therapeutics within biological systems. Additionally, there is a need for highly sensitive bioanalytical methodologies that are capable of detecting the low plasma concentrations of NBTs (Takakusa et al., 2023). Overall, the understanding of NBT delivery is expanding with recent developments focusing on increasing endosomal escape and improving bioavailability. However, challenges such as nuclease degradation, sequestration, and delivery to target cells remain, and understanding the PK and PD of NBTs is imperative for NBT development and the optimization of clinical trials.

## 6 CNS administration techniques

### 6.1 Intrathecal administration

**FIGURE 3:**
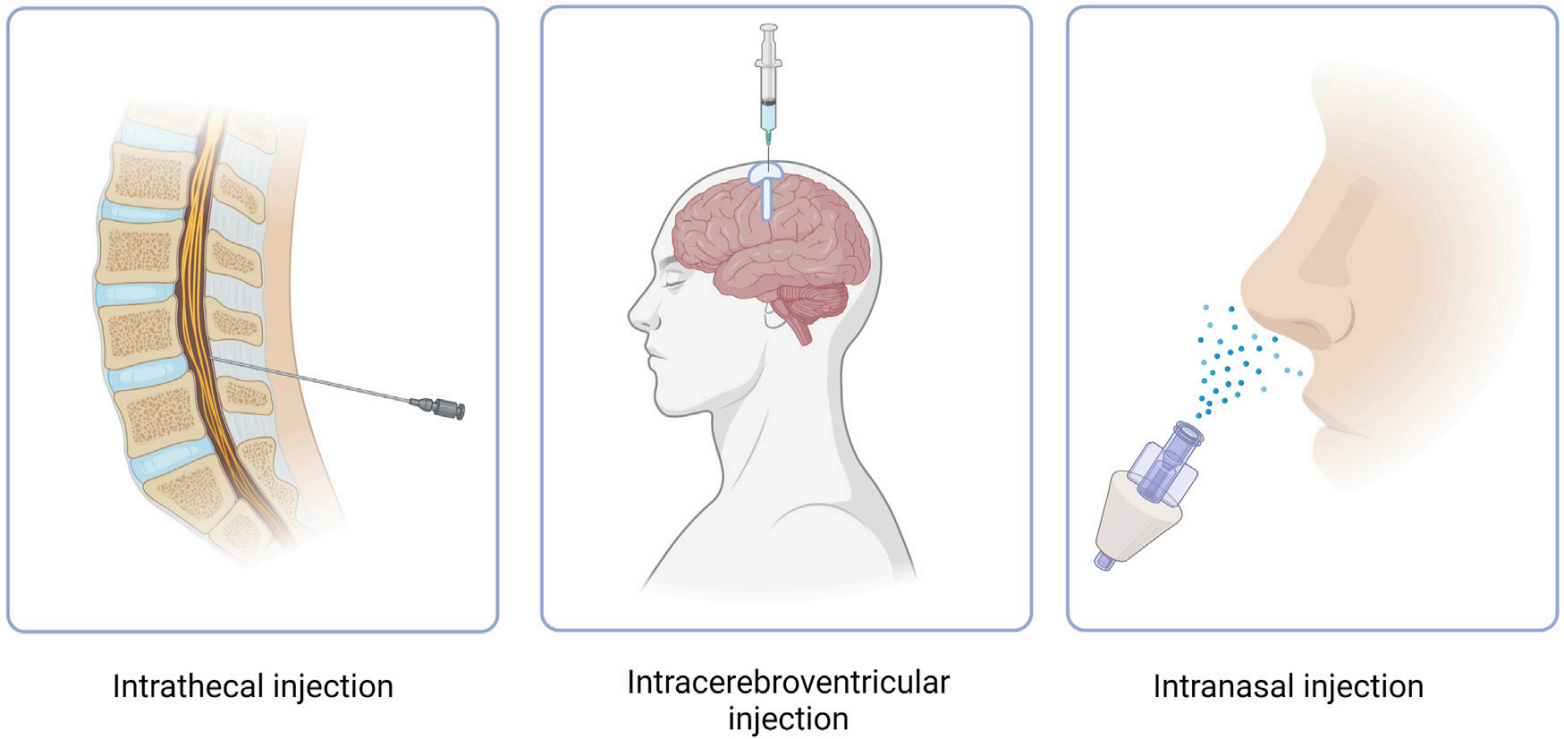
Routes of administration of NBT. Intracerebroventricular route features use of an Ommaya reservoir.

CNS targeting by both ASOs and siRNAs can be achieved via direct brain delivery through IT (intrathecal) injection (Goto et al., 2023). IT administration involves injecting the drug into the CFS within the spine and can be performed in a variety of ways, including intralaminar injection, transforaminal injection, or using a subcutaneously implanted catheter for repeat injections (Figure 3) (Lee et al., 2022; Alkosha, 2023). IT injection is the indicated route of administration for 16 oligonucleotide therapeutics that are under development as of 2022 (Goto et al., 2023) (Table 2). However, IT injections are invasive procedures and carry risks of side effects such as headaches and back pain, which does not encourage wide clinical acceptance of NBTs. Around 10% of spinal muscular atrophy patients who receive nusinersen IT injections report experiencing negative side effects after the injection (Veerapandiyan et al., 2020).

### 6.2 Intracerebroventricular administration

Another method of NBT administration that bypasses the BBB is ICV injection. While extremely invasive, ICV injections allow for drugs to directly penetrate the brain, leading to increased bioavailability and effectiveness (Figure 3) (Atkinson, 2017). In cases where repeat injections are necessary, an Ommaya reservoir can be utilized. An Ommaya reservoir allows for direct ventricular access via an intraventricular catheter system (Figure 3) (Zubair and De Jesus, 2022). A study investigating the safety and efficacy of repeat ICV injections via an Ommaya reservoir for the treatment of epilepsy has shown this administration method to be safe and effective (Cook et al., 2020). While Ommaya reservoirs have been most commonly utilized in the administration of chemotherapeutic agents, treatments for meningitis, and more currently, epilepsy, a recent study has proven the use of the reservoir to be successful in the administration of the ASO nusinersen (Iannaccone et al., 2021). In addition to an Ommaya reservoir, ventriculoperitoneal shunts can be utilized for ICV injections in patients with pre-existing shunts. By connecting an external pump or reservoir to the shunt system, drugs can be precisely and repeatedly administered into the ventricles and distributed throughout the brain (Duma et al., 2019). However, a recent study revealed that out of 111 ICV injections, approximately 11% resulted in transient meningismus and mild temperature increase, with 1.8% requiring hospitalization (Duma et al., 2019). These complications can be minimized by treatment with prophylactic dexamethasone and the use of a ventriculoperitoneal shunt in place of an Ommaya reservoir (Duma et al., 2019). Given the invasiveness and risk of side effects, ICV injections have not yet proven to be a common route of administration for NBTs.

### 6.3 Intravenous administration

A less invasive, albeit currently limited form of drug administration, is systemic administration via intravenous injection. When administered systemically, drugs must be modified to penetrate the BBB and limit off-target effects. As previously discussed, nanotechnology has been under development to promote the bypassing of the BBB. Current nanotechnologies that have shown efficacy in delivering oligonucleotide therapeutics to the brain in animal models include PLGA-coated polymeric nanoparticles, NT-lipidoids, AuNPs, and exosome delivery systems (Khongkow et al., 2019; Ma et al., 2020; Li et al., 2021; Zhang et al., 2021; Sun et al., 2022).

To augment advancements in nanotechnology that have somewhat mitigated this problem by increasing the percentage of NBT dose available to the brain after systemic delivery, novel methods of NBT administration can also play a role in bypassing the BBB.

### 6.4 Intranasal administration

Intranasal administration has recently gained attention as an attractive method for drug delivery due to its numerous advantages (Figure 3). These advantages include rapid drug delivery to the brain, reduced potential for off-target effects due to low systemic exposure, a non-invasive procedure and the feasibility of self-administration (Lochhead and Thorne, 2012). When administered intranasally, drugs reside in the nasal cavity before entering the brain through one of two pathways, the olfactory nerve pathway, or the trigeminal nerve pathway. However, drug absorption via the olfactory nerve is the most effective and best understood due to the presence of extensive epithelia and vasculature and the short length of the nerve in comparison to the trigeminal nerve (Lochhead and Thorne, 2012). The olfactory nerve pathway begins at the olfactory epithelium which is located at the upper aspect of the nasal cavity. The olfactory epithelium houses olfactory receptor neurons which absorb the drug. The axons of these neurons cross the cribriform plate, a porous bone located at the base of the skull, before reaching the olfactory bulb. From the olfactory bulb, the drug can disperse to various brain regions through neuronal connections, allowing it to reach specific target areas to exert its intended therapeutic effects (Jeong et al., 2023). Drugs can also travel through the extracellular fluid surrounding the olfactory neurons after passing through the tight junctions of epithelial cells, toward the lamina propria, a noncellular layer of connective tissue. From the lamina propria the drugs can translocate through the perineural space until the subarachnoid space is reached and further brain distribution can ensue (Crowe and Hsu, 2022).

While PK studies of intranasal administration are limited, there is evidence to suggest that NBTs administered via this route can reach the olfactory bulb as soon as 5 min post-administration and more distal regions of the brain as soon as 30 min in the mouse brain (Renner et al., 2012; Falcone et al., 2014). Nanotechnological modifications including polymeric nanoparticles and solid lipid nanoparticles can make the process of uptake more efficient and successful (Maher et al., 2023). As previously discussed, the size, surface qualities, and biocompatibility of these nanocarriers allow for efficient uptake and bypassing of the BBB (Begines et al., 2020). To this effect, a cationic lipid nanoparticle encapsulating an ASO targeting the HuR gene was shown to effectively knock down levels of HuR in the mouse brain when administered intranasally at a dose of 1 nmol (Borgonetti and Galeotti, 2021). In an additional study, the same anti-HuR ASO encapsulated in the cationic lipid nanoparticle demonstrated protection against demyelination in the mouse brain when administered intranasally (Borgonetti and Galeotti, 2023). Similar to drugs administered systemically, drugs administered intranasally can also be modified to bind to receptors present on the BBB, such as T7 siRNA modifications allowing for uptake by the transferrin receptor. In a recent study, a T7-modified siRNA targeting gliomas showed efficacy in positively altering the tumor microenvironment when administered intranasally (Tang et al., 2023).

Drug uptake via the intranasal route is most successful when the drug resides for longer periods within the nasal cavity. Considering this, modifications can be made to the drug to allow for longer residence time. One such modification is the conjugation of chitosan, a mucoadhesive agent which functions by electrostatically interacting with the negatively charged epithelial surfaces and thus enhancing the drug’s duration of stay in the nasal cavity (Maher et al., 2023). A study exploring the intranasal administration of a chitosan-based nanoparticle containing HTT targeting siRNA demonstrated therapeutically significant lowering of HTT mRNA in the mouse brain (Sava et al., 2021). Another method to increase drug residence time in the nasal cavity is through the use of biogels, which are liquids that transition to gels at body temperature (Thakkar et al., 2021). When a drug was administered intranasally in mice along with a biogel, the transport efficiency of the drug from the nasal cavity to the brain was significantly enhanced (Xie et al., 2019). The use of biogels for the intranasal administration of NBTs remains to be investigated, however.

One of the drawbacks of intranasal formulations is that the drug dose cannot be delivered accurately. A recently proposed minimally invasive intranasal depot (MIND) technique circumvents this problem by depositing ASO-containing gels under the olfactory epithelium through a simple ambulatory procedure (Padmakumar et al., 2022). MIND-mediated delivery of an ASO capable of de-repressing BDNF expression resulted in its wide CNS distribution followed by sustained upregulation of BDNF to approximately 40% of levels achieved with ICV delivery (Padmakumar et al., 2021).

Other additives that have been shown to improve intranasal delivery *in vivo* include vasoconstrictors which reduce the likelihood of the drug being absorbed into circulation, enzyme modulators which can reduce the function of enzymes that may degrade the drug, and nasal permeability enhancers which increase absorption through the nasal epithelium (Dhamankar and Donovan, 2017; Rabinowicz et al., 2021; Moradi and Dashti, 2022).

## 7 Clinical development of CNS-targeting NBTs

To date, there are two commercially available FDA-approved NBTs for CNS disorders (Table 1). Nusinersen (Spinraza), approved in 2016 for the treatment of SMA, was the first NBT to gain FDA approval and has since shown success in improving functional outcomes and slowing disease progression (Pechmann et al., 2022). SMA is caused by a mutation in the SMN1 gene which leads to a deficiency in the SMN protein that is vital for motor neuron function. Nusinersen functions by modifying the splicing process of an inactive duplicate gene, SMN2, resulting in a transcript that codes for the functional SMA protein (Neil and Bisaccia, 2019).

**TABLE 1:** NBTs approved by FDA for CNS disorders *Not commercially available.

Condition	Drug name	Target	Delivery method	Manufacturer	Year approved
Spinal muscular atrophy	Nusinersen (Spinraza)	SMN2	IT	Ionis/Biogen	2016
Batten’s disease	Milasen*	ATXN2	IT	Boston Children’s Hospital	2018
Amyotrophic lateral sclerosis	Tofersem (Qalsody)	SOD1	IT	Ionis/Biogen	2023

After the initial loading phase, nusinersen is administered to patients via IT injection every 4 months (Zingariello et al., 2019). Adverse effects of nusinersen treatment are rare and usually limited to side effects from the IT injection (Neil and Bisaccia, 2019). Despite being a very effective treatment option, treatment with nusinersen remains inaccessible to many patients due to the high price. A recent study demonstrated that of 324 patients, 50% discontinued treatment after 12 months. Non-adherence was correlated with greater frequency of comorbidities and increased costs for patients (Gauthier-Loiselle et al., 2021).

The second, and most recently approved NBT for a CNS disorder, is Qalsody (tofersen). Tofersen was approved in April 2023 for the treatment of amyotrophic lateral sclerosis (ALS) associated with mutations in the SOD1 gene (Hara Prasad, 2023). Mutations in the SOD1 gene can lead to both toxic gain of function and toxic loss of function in mutant SOD1 proteins, leading to the damage of motor neurons (Berdynski et al., 2022). Tofersen functions by mediating the RNAse-H degradation of the pathogenic SOD1 mRNA transcript which reduces levels of the toxic SOD1 mutant protein (Miller et al., 2020). While the functional outcomes of patients treated with tofersen did not differ from patients treated with a placebo, 28 weeks of once-monthly IT injections did lead to a significant decrease in the total plasma concentration of SOD1 (Miller et al., 2022).

Another NBT, Milasen, was approved by the FDA in 2018 for the treatment of Batten’s disease in a single patient. Milasen functions by modulating the splicing of the gene MFSD8 and was administered to the patient via IT injection (Kim et al., 2019). While specific to only one patient and not commercially available, the development and subsequent FDA approval of milasen serves as a proof of concept for the potential of “n of 1” NBT therapeutics.

Several other CNS targeting NBTs are now in late stages of development (Table 2). In April 2023 Ionis announced positive results of eplontersen study in hereditary ATTR polyneuropathy. At 66 weeks, the study met all endpoints, including reduction in serum TTR concentration and neuropathy impairments, as well as improvements in quality of life (Ionis Pharmaceuticals, 2023).

**TABLE 2:** NBTs in clinical trials for CNS disorders.

Condition	Drug name	Target	Delivery method	Sponsor name	Phase	ClinicalTrials.gov ID
Amyotrophic lateral sclerosis	Ulefnersen (ION363)	FUS	IT	Ionis	III	NCT04768972
Amyotrophic lateral sclerosis	ION541 (BIIB105)	ATXN2	IT	Ionis/Biogen	I/II	NCT04494256
Huntington’s disease	AMT-130	HTT	Intra-striatal injection	UniQure Biopharma	I/II	NCT04120493
Huntington’s disease	Tominersen (IONIS-HTTRx, RG6042)	HTT	IT	Ionis/Roche	II	NCT05686551, NCT04000594, NCT03842969
Huntington’s disease	WVE-003	HTT	IT	Wave Life Sciences	I/II	NCT05032196
Hereditary ATTR polyneuropathy	Eplontersen (IONIS-TTR-LRx, AKCEA-TTR-LRx)	HTT	Self-administered SC	Ionis/Astra Zeneca	III	NCT05667493, NCT05071300, NCT04136171
Alexander disease	Zilganersen (ION373)	GFAP	IT	Ionis	III	NCT04849741
Alzheimer’s disease	IONIS-MAPTRx (BIIB080)	MAPT	IT	Ionis/Biogen	II	NCT03186989, NCT02831517, NCT02957617
Alzheimer’s disease, early onset	ALN-APP	APP	IT	Alnylam/Regeneron	I/II	NCT05231785
Dravet syndrome	STK-001	SCN1A	IT	Stoke Therapeutics	II	NCT04740476
Parkinson’s disease	ION859 (IONIS-BIIB7Rx, BIIB094)	LRRK2	IT	Ionis/Biogen	I/II	NCT03976349
Angelman syndrome	ION582	UBE3A-ATS	IT	Ionis/Biogen	I/II	NCT05127226
Multiple system atrophy	ION464 (BIIB101)	SNCA	IT	Ionis	I/II	NCT04165486

IT, intrathecal injection; SC, subcutaneous injection.

At the same time Alnylam and Regeneron reported positive results from the trial of ALN-APP, an siRNA NBT against the amyloid precursor protein (APP). CSF concentrations of both soluble APPα and its metabolite Abeta were reduced by up to 70% for at least 3 months at the highest dose tested (Alnylam Pharmaceuticals, 2023).

Treatment of AD patients with another ASO, IONIS-MAPTRx, resulted in a dose-dependent reduction in the CSF concentration of protein tau of more than 50% from baseline at 24 weeks after 2 or 4 doses of IONIS-MAPTRx (Mummery et al., 2023).

Wave Life Sciences is developing WVE-003, a stereopure ASO that targets a specific single nucleotide polymorphism (SNP3) within the huntingtin gene, allowing for specific destruction of transcripts with expanded repeat sequence. Preliminary results of the clinical trial announced in September 2022 showed media mutant HTT reduction of 30% from baseline at 85 days post single IT injection (Wave Life Sciences USA, 2022).

Other trials are on-going (Table 2), indicating a significant interest in developing NBTs for CNS disorders.

## 8 Current challenges and future perspectives in CNS-targeting NBTs

While NBTs have proven to be a promising treatment option for CNS diseases, there remain challenges that need to be addressed. Major challenges include effectively bypassing the BBB, improving tissue and cell-specific targeting, and enhancing cellular uptake and endosomal escape.

The development of nanocarriers has helped address the challenge of bypassing the BBB due to their small size, biocompatibility, and ability for surface functionalization (Amulya et al., 2023). Targeting extrahepatic ligands such as the transferrin receptor may be a promising avenue for brain-specific uptake. NBTs conjugated to Tfr1 binding moieties and anti-TfR antibodies have shown preclinical success (Barker et al., 2023; Gabold et al., 2023).

In regard to cell-specific targeting, methods to specifically target glial cells or neurons remain to be elucidated. This is especially important as glial cells, such as microglia, play a pivotal role in the pathology of many CNS diseases.

Cellular uptake of NBTs has been enhanced through chemical modifications and the development of nanotechnologies (Crooke et al., 2017). Endosomal escape, however, remains a pertinent issue as endosomes may retain as much as 99% of NBTs, leaving only 1% of the drug to exert therapeutic effects (Dowdy, 2023). Technologies that promote endosomal escape remain under development, but the incorporation of cell-penetrating peptides or sphingolipid treatment may be promising (Dastpeyman et al., 2021).

Furthermore, the understanding of brain distribution of NBTs remains limited and requires further elucidation. Future research into this area should include investigation of the molecular mechanisms governing the brain distribution of NBTs, physiological and anatomical differences affecting brain NBT distribution, and quantification of intracellular NBT distribution (D’Souza et al., 2023). Understanding these principles will aid in the development of future CNS-targeted NBTs. For example, in regard to the treatment of Parkinson’s disease, the target dopaminergic neurons of the substantia nigra and ventral tegmental area comprise only 600,000 cells (Holm et al., 2022). Precise targeting of this small subset of neurons will reduce the required dose and possibility off-target effects of NBTs.

Lastly, the administration methods of NBTs targeting the CNS remain a challenge, with a need for a non-invasive and effective method for bypassing the BBB. The majority of brain targeting NBTs are currently administered via IT injection (Goto et al., 2023). While effective, IT administration proves invasive and is not easily accessible to all patients. Alternative administration routes such as intranasal administration are currently being explored and hold significant potential (Maher et al., 2023).
